# A new insight into high-strength Ti_62_Nb_12.2_Fe_13.6_Co_6.4_Al_5.8_ alloys with bimodal microstructure fabricated by semi-solid sintering

**DOI:** 10.1038/srep23467

**Published:** 2016-03-31

**Authors:** L. H. Liu, C. Yang, L. M. Kang, S. G. Qu, X. Q. Li, W. W. Zhang, W. P. Chen, Y. Y. Li, P. J. Li, L. C. Zhang

**Affiliations:** 1 National Engineering Research Center of Near-net-shape Forming for Metallic Materials, South China University of Technology, Guangzhou 510640, China; 2 Department of Mechanical Engineering, Tsinghua University, Beijing 100084, China; 3 School of Engineering, Edith Cowan University, 270 Joondalup Drive, Joondalup, Perth, WA 6027, Australia

## Abstract

It is well known that semi-solid forming could only obtain coarse-grained microstructure in a few alloy systems with a low melting point, such as aluminum and magnesium alloys. This work presents that semi-solid forming could also produce novel bimodal microstructure composed of nanostructured matrix and micro-sized (CoFe)Ti_2_ twins in a titanium alloy, Ti_62_Nb_12.2_Fe_13.6_Co_6.4_Al_5.8_. The semi-solid sintering induced by eutectic transformation to form a bimodal microstructure in Ti_62_Nb_12.2_Fe_13.6_Co_6.4_Al_5.8_ alloy is a fundamentally different approach from other known methods. The fabricated alloy exhibits high yield strength of 1790 MPa and plastic strain of 15.5%. The novel idea provides a new insight into obtaining nano-grain or bimodal microstructure in alloy systems with high melting point by semi-solid forming and into fabricating high-performance metallic alloys in structural applications.

Nanostructured materials often exhibit low ductility at room temperature and very limited or lack of work hardening due to limited dislocation capability^1^. One feasible and practical approach to enhance the ductility of nanostructured materials is to form bimodal microstructure with coexistence of nanoscale and micron-sized grains^2^. The formation of such a bimodal microstructure could provide nanostructured materials with both high strength and ductility compared to single-phase nanostructured materials or conventional coarse-grained materials^3^. In general, such bimodal microstructure can be obtained by several routes, such as thermo-mechanical treatment^4^, powder consolidation^5^, recrystallization method^6^, and rapid solidification^7^. Especially, many titanium alloys with bimodal microstructure have been prepared by rapid solidification and exhibits high strength and large plasticity^7^,^8^,^9^,^10^,^11^,^12^,^13^. For example, the Ti_60_Cu_14_Ni_22_Sn_4_Nb_10_ alloy has strength of 2400 MPa and plastic strain of 14.5%^7^ and Ti_63.375_Fe_34.125_Sn_2.5_ exhibits strength of 2650 MPa and a plasticity of 12.5%^12^. The typical metallurgical characteristics for obtaining bimodal-microstructure titanium alloys by rapid solidification are preferential nucleation and growth of micron-sized body-centered cubic (bcc) β-Ti dendrites from high-temperature melts followed by rapid solidification of the remaining liquid with highly dense random-packed structure to obtain nanostructured matrix^7^,^8^,^9^,^10^,^11^,^12^,^13^.

As one of the important materials processing technologies, the core feature of semi-solid forming includes special non-dendrite solid microstructure and moderate forming temperature locating between solidus and liquidus temperature^14^. Integrated across multi-disciplines, a series of semi-solid forming methods, coupling with casting, extrusion, forging, rolling, and so on, have been developed spontaneously. However, current semi-solid forming usually includes a relatively complicated process for preparing semi-solid alloy slurry, and it unfortunately can only produce coarse-grained microstructure in a few alloy systems with a low melting point^15^, such as aluminum alloys and magnesium alloys. So far, it is impossible to form nanocrystalline or bimodal microstructure in alloy systems having a high melting point, for example titanium alloys.

According to binary alloy phase diagrams^16^, a typical eutectic transformation occurred at eutectic temperature can be expressed as *α* + *β* ↔ *L*, where *α* and *β* are two solid components and *L* is a liquid state. Currently, the liquid phase in semi-solid forming based on eutectic transformation has a relatively loose random-packed structure in aluminum alloys and magnesium alloys^17^. This is the reason why current semi-solid forming cannot obtain nanocrystalline or bimodal microstructure. As such, a question arises: if a liquid phase, resulted from preferential eutectic reaction of two solid phases in a multi-phase alloy system with a high melting point, has a highly dense random-packed structure, does the composition of this liquid phase tend to form a nanostructured phase/microstructure in process of solidification^17^,^18^? Undoubtedly, the semi-solid state consisting of such a liquid phase is different from those in the aforementioned semi-solid forming. As supported by the extensive studies (e.g. refs 17,18) on the formation of nanostructure or glassy alloys by rapid solidification, a semi-solid state with highly dense random-packed structure is easy to be obtained in multicomponent alloy systems having a high melting point. Such a semi-solid state characteristic with highly dense random-packed structure may be of significance and could be employed for fabricating new-structure materials, such as with nano-grained or bimodal microstructure, by semi-solid forming from multicomponent alloy systems having a high melting point. This could break through the bottleneck of current semi-solid forming, i.e. producing coarse-grained microstructure from alloys with low melting point, and improve its capability for processing alloys with a high melting temperature and for forming novel microstructure as well.

In this work, based on the aforementioned assumption, a material forming method coupling sintering nanocomposite powder with subsequent semi-solid treatment induced by eutectic transformation, referred to as semi-solid sintering, was introduced to fabricate bimodal microstructure in a Ti_62_Nb_12.2_Fe_13.6_Co_6.4_Al_5.8_ alloy engineered for both high strength and large ductility. The as-fabricated bimodal microstructure is different from those reported so far, as examples in refs 4, 5, 6, 7, 8, 9, 10, 11, 12, 13. The novel idea is expected to give some insight into fabricating nanostructured metallic alloys having high melting point for high-performance structural applications.

## Results and Discussion

**Figure 1:**
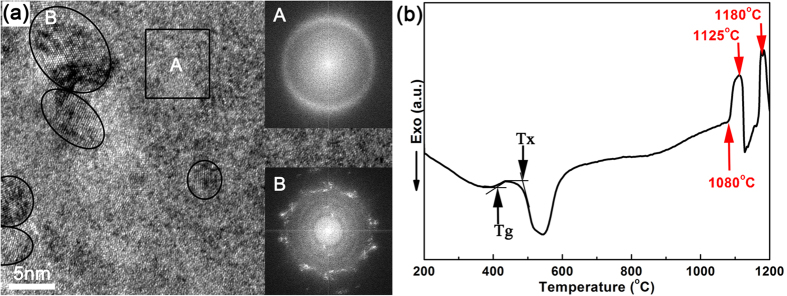
(**a**) High-resolution TEM image and (**b**) DSC curve of the 70 h-milled Ti_62_Nb_12.2_Fe_13.6_Co_6.4_Al_5.8_ powder. The inset A and B in (**a**) are the Fourier-transformed images for the square area and ellipse area, respectively, displaying that the 70-milled powder contains mainly nano-sized β-Ti surrounded by glassy matrix. Two evident endothermic peaks in (**b**) at temperatures of 1125 °C and 1180 °C confirm the existence of a semi-solid interval between 1080–1200 °C in this alloy.

Figure 1a shows a high-resolution TEM image and the Fourier-transformed images for the square area and ellipse area of the 70 h-milled Ti_62_Nb_12.2_Fe_13.6_Co_6.4_Al_5.8_ powder, i.e. the starting powder used for semi-solid sintering. As seen from Fig. 1a, the 70-milled powder consists mainly of nano-sized β-Ti surrounded by glassy matrix. Its glassy nature is further confirmed by the clear endothermic glass-transition event (at 420 °C) and the strong exothermic crystallization peak (at 490 °C) on the DSC curve of the 70 h-milled powder (Fig. 1b). Two evident endothermic peaks are observed at temperatures of 1125 °C and 1180 °C respectively for the 70 h-milled Ti_62_Nb_12.2_Fe_13.6_Co_6.4_Al_5.8_ nanocomposite powder, confirming that there exists a semi-solid interval between 1080 °C and 1200 °C for the Ti_62_Nb_12.2_Fe_13.6_Co_6.4_Al_5.8_ alloy.

**Figure 2:**
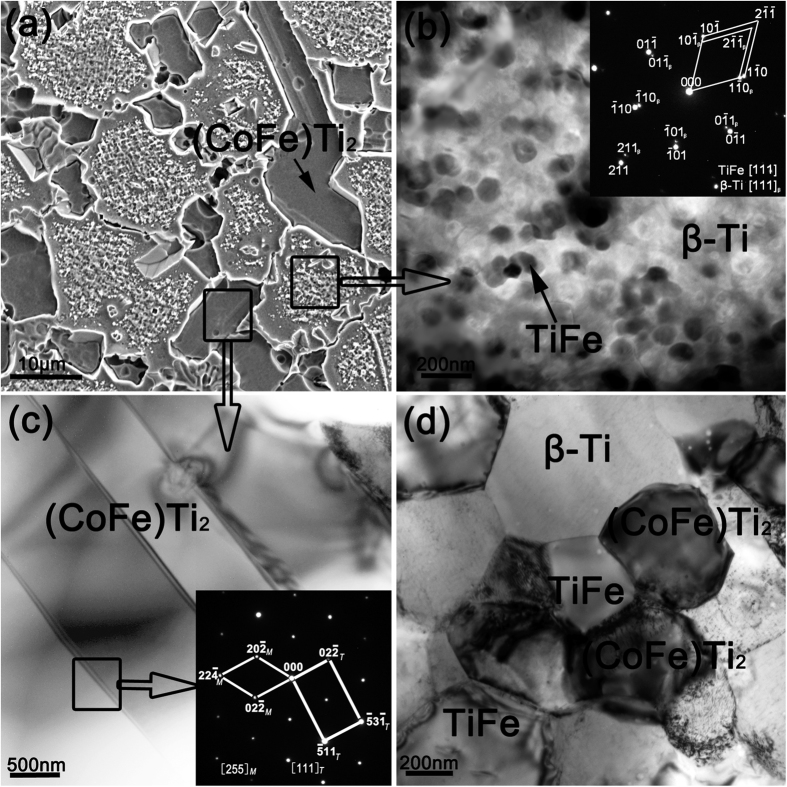
(**a**) SEM microstructure of the semi-solid sintered alloy at 1100 °C. (**b**) TEM image of composite structure matrix, the inset displaying corresponding SAD patterns of the matrix. (**c**) TEM image and corresponding SAD pattern of fcc (CoFe)Ti_2_ twins. (**d**) TEM image of the alloy sintered at 950 °C.

Figure 2a shows the SEM microstructure of the semi-solid sintered Ti_62_Nb_12.2_Fe_13.6_Co_6.4_Al_5.8_ alloy at 1100 °C. Interestingly, the sample consists of a lath-shaped (CoFe)Ti_2_ twins with a size scale of several tens of microns dispersed in a complicated matrix (Fig. 2a). Detailed TEM analysis (Fig. 2b) of the matrix proves a microstructure of equiaxed TiFe particles with a grain size of 80–120 nm surrounded by β-Ti phase. The corresponding selected-area diffraction (SAD) pattern taken along the [111] zone axis for both β-Ti and TiFe (the inset in Fig. 2b) confirms that the TiFe particle has a bcc CsCl-type structure with a lattice parameter of 0.2998 nm, which is close to that of bcc β-Ti phase (0.3205 nm). The (CoFe)Ti_2_ twins display an inter-lath spacing of 500–1000 nm (Fig. 2c). The average chemical compositions of the (CoFe)Ti_2_, TiFe and β-Ti phases determined by EDX are Ti_62.02_Nb_2.77_Fe_17.40_Co_17.38_Al_2.41_, Ti_58.17_Nb_4.78_Fe_31.19_Co_4.75_Al_4.78_ and Ti_64.92_Nb_25.85_Fe_4.47_Co_0.32_Al_4.43_, respectively. In this case, the obtained bimodal microstructure, i.e. the coexistence of nanostructured matrix and micron-sized (CoFe)Ti_2_ twins, is different from those in the samples fabricated by thermo-mechanical treatment, powder consolidation, recrystallization method, or rapid solidification^4^,^5^,^6^,^7^,^8^,^9^,^10^,^11^,^12^,^13^. In contrast, the sample sintered at 950 °C, not undergoing semi-solid treatment, is composed of equiaxed face-centered cubic (fcc) (CoFe)Ti_2_ and bcc TiFe phases with grain sizes of 200–400 nm embedded into equiaxed β-Ti matrix with a grain size of 400–600 nm (Fig. 2d). This equiaxed microstructure is similar to that in titanium alloys by solid-phase sintering of nanocomposite powders in our previous works^19^,^20^,^21^,^22^.

**Figure 3:**
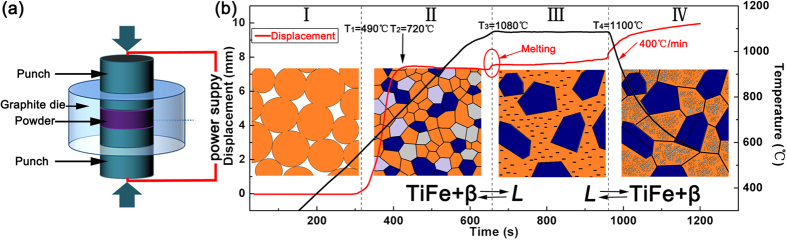
(**a**) Schematic of sintering experiment and (**b**) the recorded shrinkage displacement of the punch as well as the measured temperature as a function of the sintering time. The inset diagrams in (**b**) show the microstructure evolution at different stages. “*L*” and “β” denote liquid phase and β-Ti phase, respectively.

In order to elaborate the formation mechanism of the bimodal microstructure in titanium alloys during semi-solid sintering, the microstructure evolution in the course of the whole sintering is schematically presented in Fig. 3. As seen from the recorded shrinkage displacement of the punch and the measured temperature as a function of the sintering time (Fig. 3b), the semi-solid processing can be basically divided into four stages. At stage I (temperature below ~490 °C), there is no significant increase in the punch displacement, implying that the rearrangement of powder particles dominates the densification process^23^. At stage II (temperature ranging 490-1080 °C), the punch displacement rapidly increases first and then stabilizes at a temperature threshold of 720 °C. Below 720 °C, the increase in punch displacement is attributed to that the TiFe and (CoFe)Ti_2_ phases begin to nucleate and grow from glassy matrix or supersaturated β-Ti phase in sintered nanocomposite powder, leading to a rapid densification^21^. Above 720 °C, the densification process has completed but grain growth of the three constituent phases (i.e. β-Ti, TiFe, and (CoFe)Ti_2_) continues until forming liquid phase. At stage III (temperature locating at semi-solid temperature interval), the punch displacement displays an instantaneous increase around 1080 °C, indicating the formation of liquid phase induced by the eutectic transformation between the β-Ti and TiFe. This is in good agreement with the onset melting temperature (~1080 °C) on the DSC curve (Fig. 1b) of the as-milled Ti_62_Nb_12.2_Fe_13.6_Co_6.4_Al_5.8_ nanocomposite powder. It should be noted that the sintering pressure is relieved immediately once liquid phase forms. Before depressurization, the deformation effect resulting from sintering pressure leads to the formation of fcc (CoFe)Ti_2_ twins (Fig. 2c). Afterwards, the semi-solid state containing the as-formed liquid phase and remaining solid (CoFe)Ti_2_ twins is holding at constant 1100 °C under pressure relief. Finally, at stage IV, the as-formed liquid phase is solidified to form the nanostructured matrix at a cooling rate of 400 °C/min (~6.6 K/s) (Fig. 3b), which is far below the magnitude order of the cooling rate (about 100 ~ 1000 K/s) for forming bimodal nanostructure-dendrite titanium alloys by rapid solidification in a water-cooled copper mould^7^,^8^,^9^,^10^,^11^,^12^,^13^. The reason of forming such a bimodal microstructure composed of nanostructured matrix and micro-sized (CoFe)Ti_2_ twins in the Ti_62_Nb_12.2_Fe_13.6_Co_6.4_Al_5.8_ alloy is mainly ascribed to the highly dense random-packed structure of the remaining liquid in the multicomponent TiNbFeCoAl alloy system. It is well known that a multicomponent glass forming alloy usually has more than three kinds of constituent elements with large atomic size ratios and negative heats of mixing^17^,^24^. This can induce a highly dense random-packed structure in the liquid from technological and chemical points of view^17^,^24^. Moreover, glass forming ability or degree of dense random packed structure for a multicomponent alloy can be further enhanced by proper adding or modifying elements^24^. The two aspects are contributed to the formation of the solidified nanostructured matrix from the remaining liquid in the present modified multicomponent TiNbFeCoAl alloy by the addition of refractory element Nb^7^,^8^,^9^. In a word, the formation mechanism of the bimodal microstructure in this work is reversible to that in rapid solidification^7^,^8^,^9^,^10^,^11^,^12^,^13^. Compared to the formation of bimodal microstructure by rapid solidification, the similar feature is the preferential formation of micron-sized solid microstructure under semi-solid state and the subsequent formation of nanostructured matrix by solidifying the remaining liquid phase.

Theoretically and experimentally, forming liquid phase in the semi-solid forming in this work can be explained based on the eutectic transformation between the β-Ti and TiFe phases. According to the Fe-Ti and Co-Ti binary phase diagrams^16^, there exist two eutectic reactions at 1085 °C for bcc TiFe and bcc β-Ti and at 1020 °C for fcc CoTi_2_ and bcc β-Ti, respectively, to transform into liquid phase. Two melting endothermic peaks with peak temperatures of 1125 °C and 1180 °C respectively are distinctly observed on the DSC curve (Fig. 1b) for the as-milled Ti_62_Nb_12.2_Fe_13.6_Co_6.4_Al_5.8_ powder. This confirms the existence of a semi-solid state locating between 1080 °C and 1200 °C in the Ti_62_Nb_12.2_Fe_13.6_Co_6.4_Al_5.8_ alloy. The theoretical eutectic temperature of 1085 °C for bcc TiFe and bcc β-Ti is well coincident with the onset melting temperature of 1080 °C obtained from the DSC analysis (Fig. 1b) and the exact temperature of instantaneous increase in the punch displacement during sintering process (Fig. 3). This proves that the formation of liquid phase may be attributed to the eutectic transformation between the β-Ti and TiFe phases. In this case, the sample sintered at 950 °C has three phases, i.e. fcc (CoFe)Ti_2_, bcc TiFe and bcc β-Ti. Note that the (CoFe)Ti_2_ is a solid solution of Fe atoms substituting for Co positions in fcc CoTi_2_ compound. The solution of Fe atoms may enhance largely the eutectic transformation temperature of the fcc (CoFe)Ti_2_ and bcc β-Ti^10^. Consequently, the bcc TiFe and bcc β-Ti react preferentially into liquid phase and the fcc (CoFe)Ti_2_ remains solid state and grows from ultrafine grains (Fig. 2d) into micron-sized twins (Fig. 2a,c). The twin structure, usually formed and remained by deformation effect in an fcc solid phase^25^, again proves the preferential eutectic transformation between the β-Ti and TiFe.

In order to further elaborate the formation mechanism of the present novel bimodal microstructure, the Ti_62_Nb_12.2_Fe_13.6_Co_6.4_Al_5.8_ alloy was prepared by rapid solidification in a water-cooled copper mould for comparison. Obviously, the as-solidified alloy consists of only β-Ti phase and TiFe phase. It displays a typical bimodal microstructure of a primary micron-sized β-Ti phase (in a dark color) dispersed in a nanostructured eutectic matrix with β-Ti phase and TiFe phase (in a light color) (Fig. S1). This once again confirms that the formation of the semi-solid state in the present work is induced by the eutectic transformation between the β-Ti and TiFe phases rather than that between the β-Ti and (CoFe)Ti_2_ phases. Besides, the semi-solid state induced by preferential eutectic transformation between the β-Ti and TiFe rather than by that between the β-Ti and (CoFe)Ti_2_ can also be supported by the additional experiments carried out in Ti_66_Nb_13_Fe_8_Co_6.8_Al_6.2_ alloy with lower Fe content (Figs S2–5 in the supplementary information compared with the present Ti_62_Nb_12.2_Fe_13.6_Co_6.4_Al_5.8_ alloy). As seen from Fig. S2, single melting endothermic peak with a peak temperature of 1185 °C in DSC curve, the presence of the same (CoFe)Ti_2_ twins and β-Ti in sintered Ti_66_Nb_13_Fe_8_Co_6.8_Al_6.2_ alloy (Figs S3 and S4), and absence of instantaneous increase in the punch displacement around 1080 °C (Fig. S6) during sintering for the Ti_66_Nb_13_Fe_8_Co_6.8_Al_6.2_ powder further confirm that the (CoFe)Ti_2_ phase is in a solid state at sintering temperature of 1100 °C. As such, the formation of such a liquid phase results from the preferential eutectic transformation between the β-Ti and TiFe in the Ti_62_Nb_12.2_Fe_13.6_Co_6.4_Al_5.8_ alloy. From another point of view, if the formation of liquid phase originates from a ternary eutectic transformation of the bcc β-Ti, bcc TiFe and fcc (CoFe)Ti_2_, it is impossible to obtain the bimodal microstructure in the present case according to the formation mechanism of bimodal microstructure in rapid solidification^7^,^8^,^9^,^10^,^11^,^12^,^13^. In a word, forming liquid phase in Ti_62_Nb_12.2_Fe_13.6_Co_6.4_Al_5.8_ alloy is certainly attributed to the preferential eutectic transformation between the β-Ti and TiFe phases.

**Figure 4:**
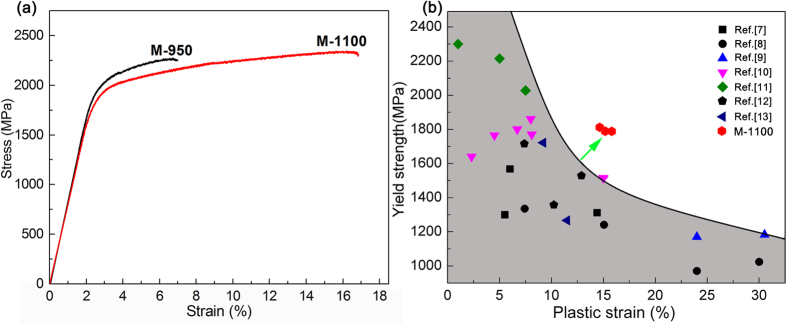
(**a**) Engineering stress-strain curves for the semi-solid sintered Ti_62_Nb_12.2_Fe_13.6_Co_6.4_Al_5.8_ alloys. The “X” in the sample “M-X” denotes the sintering temperature, (**b**) Yield strength versus plastic strain of the titanium alloys reported so far with a bimodal microstructure.

**Figure 5:**
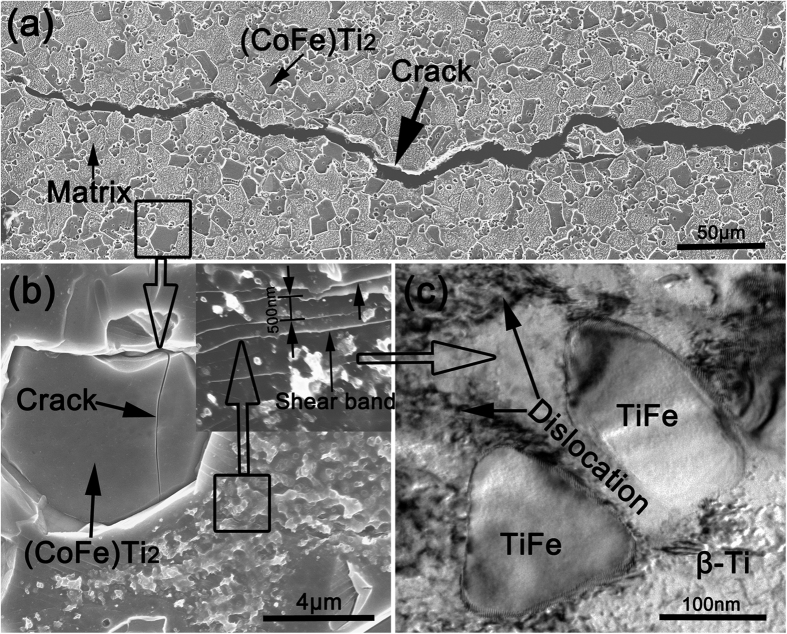
(**a**) SEM micrograph of fractured surface, displaying that macroscopic crack propagates along with the boundaries of micron-sized fcc (CoFe)Ti_2_ twins and nanostructured matrix or passes through micron-sized fcc (CoFe)Ti_2_ twins. (**b**) Magnified detail information of local deformed region, presenting that the cracks were produced preferentially in fcc (CoFe)Ti_2_ twins and hindered by the matrix. The inset shows that profuse of shear bands formed in the matrix and blocked by nano-sized TiFe particles, implying excellent strain-hardening capacity. (**c**) TEM image observed in local deformed matrix, indicating that lots of dislocations generated in β-Ti regions were blocked by TiFe particles.

Figure 4a presents the compressive engineering stress-strain curve for the semi-solid sintered Ti_62_Nb_12.2_Fe_13.6_Co_6.4_Al_5.8_ alloy compared with the sintered sample at 950 °C. All the mechanical properties, i.e. strength and strain in deformation, were averaged by three tests. As seen from Fig. 4a, the equiaxed-grained sample sintered at 950 °C exhibits ultrahigh yield strength of 1850 MPa but limited plastic strain. In contrast, the sample with bimodal microstructure prepared by semi-solid sintering has ultrahigh yield strength of 1790 MPa as well as large plastic strain of 15.5%. In order to compare the mechanical properties of the present samples with those of other types of representative bimodal-microstructure titanium alloys by rapid solidification^7^,^8^,^9^,^10^,^11^,^12^,^13^, yield strength versus plastic strain are summarized and presented in Fig. 4b. Evidently, the semi-solid sintered samples reported herein exhibit simultaneous high strength and large plastic strain, which are superior to most of as-reported bimodal titanium by rapid solidification. This is ascribed to the collaborative effects of the three constituted phases in the bimodal microstructure under loading^12^. Profuse dislocations and shear bands formed in the β-Ti regions are blocked and branched and multiplied by the micron-sized (CoFe)Ti_2_ twins (Fig. 5b,c) and nano-sized TiFe particles (Fig. 5c). This indicates that the deformability of (CoFe)Ti_2_ phase and TiFe phase is not large. The preferentially formed micro-cracks in the isolated fcc (CoFe)Ti_2_ twins are separated, hindered and restricted by the surrounded nanostructured matrix (Fig. 5b), causing zigzag crack paths and avoiding shearing-off through the whole sample (Fig. 5a).

In summary, this work has successfully prepared a bimodal microstructure in Ti_62_Nb_12.2_Fe_13.6_Co_6.4_Al_5.8_ alloy via a novel approach of semi-solid sintering induced by eutectic transformation. Its high yield strength results from nanostructured matrix and strengthening effect of micron-sized (CoFe)Ti_2_ twins. The large plasticity is ascribed to the profuse shear bands in nano-sized β-Ti phase and associated block effects by nano-sized TiFe phase. The findings will help guide endeavors to obtain high-performance metallic materials in alloy systems with a high melting point.

## Methods

In present experiments, the process started by preparing Ti_62_Nb_12.2_Fe_13.6_Co_6.4_Al_5.8_ (at.%) alloy powder from respective element powder by mechanical alloying in a high-energy planetary ball mill (QM-2SP20, apparatus factory of Nanjing University) under a purified argon gas atmosphere. Approximately 3 g of the powder was removed from the mill-vial every 10 h for X-ray diffraction (XRD) examination (D/MAX-2500/PC, Rigaku Corp., Tokyo, Japan) until the formation of nanocomposite structure with an amorphous matrix surrounding β-Ti nanocrystals after 70 h milling. Then, differential scanning calorimetry (DSC, Netzsch STA 409 C) was employed to determine the semi-solid temperature interval of the as-milled powder. Afterwards, the as-milled powder was semi-solid sintered by the following two-step method under continuous heating to semi-solid interval (1100 °C) under an argon atmosphere by a Dr. Sintering SPS-825 system. In the first step, samples were heated from room temperature to 1050 °C at a heating rate is 100 °C/min and the sintering pressure was 50 MPa. In the second one, when the samples were heated to 1100 °C from 1050 °C at 50 °C/min under 50 MPa, the sintering pressure was relieved immediately and the sample were hold at 1100 °C for 5 min without pressure. For comparison, additional Ti_62_Nb_12.2_Fe_13.6_Co_6.4_Al_5.8_ specimens were sintered at 950 °C using the same parameters. The detailed experimental procedures can be seen in ref. 19,20.

Instantaneous values of sintering parameters, such as temperature, punch displacement, and time, were recorded every 2 seconds by the attached software. The cooling rate of the semi-solid sintered alloy was determined by calculating the slope of the temperature curve of the as-sintered alloy during the cooling process. All sintered bulk samples had a cylindrical shape with a dimension of Ф20 × 10 mm. A Philips XL-30 FEG scanning electron microscopy (SEM; Amsterdam, The Netherlands) and a Tecnai G2 F30 field emission gun transmission electron microscopy (TEM; FEI, Eindhoven, The Netherlands) coupled with energy dispersive X-ray (EDX) analysis were used for microstructure investigation. In order to evaluate the mechanical properties under compression for comparison with the reference alloys, cylindrical specimens of 3 mm in diameter and 6 mm in length were tested in a universal testing machine (MTS testing system) under quasistatic loading at a strain rate of 5 × 10^−4^ s^−1^.

## Additional Information

**How to cite this article**: Liu, L. H. *et al.* A new insight into high-strength Ti_62_Nb_12.2_Fe_13.6_Co_6.4_Al_5.8_ alloys with bimodal microstructure fabricated by semi-solid sintering. *Sci. Rep.* **6**, 23467; doi: 10.1038/srep23467 (2016).

## Supplementary Material

Supplementary Information
