# Supplementary material for: A new insight into high-strength Ti62Nb12.2Fe13.6Co6.4Al5.8 alloys with bimodal microstructure fabricated by semi-solid sintering
Source: Sci Rep. 2016 Mar 31;6:23467. doi: 10.1038/srep23467 (PMC4814828; doi:10.1038/srep23467)
Supplement: Supplementary Information [file srep23467-s1.pdf]

Supplementary information

A new insight into high-strength $Ti_{62}Nb_{12.2}Fe_{13.6}Co_{6.4}Al_{5.8}$ alloys with bimodal microstructure fabricated by semi-solid sintering

L.H. Liu[2], C. Yang[1], L.M. Kang[1], S.G. Qu[1], X.Q. Li[1], W.W. Zhang[1], W.P. Chen[1], Y.Y. Li[1], P.J. Li[2] & L.C. Zhang[3]

[1]*National Engineering Research Center of Near-net-shape Forming for Metallic Materials, South China University of Technology, Guangzhou 510640, China*

[2]*Department of Mechanical Engineering, Tsinghua University, Beijing 100084, China*

[3]*School of Engineering, Edith Cowan University, 270 Joondalup Drive, Joondalup, Perth, WA 6027, Australia*

Contents:

Fig. S1 SEM microstructure of $Ti_{62}Nb_{12.2}Fe_{13.6}Co_{6.4}Al_{5.8}$ alloy fabricated by rapid solidification in a water-cooled copper mould. It is obvious that the rapidly-solidified alloy consists of only β-Ti phase and TiFe phase. It displays a typical bimodal microstructure of a primary micron-sized β-Ti phase (in a dark color) dispersed in a nanostructured eutectic matrix with the β-Ti and TiFe phases.

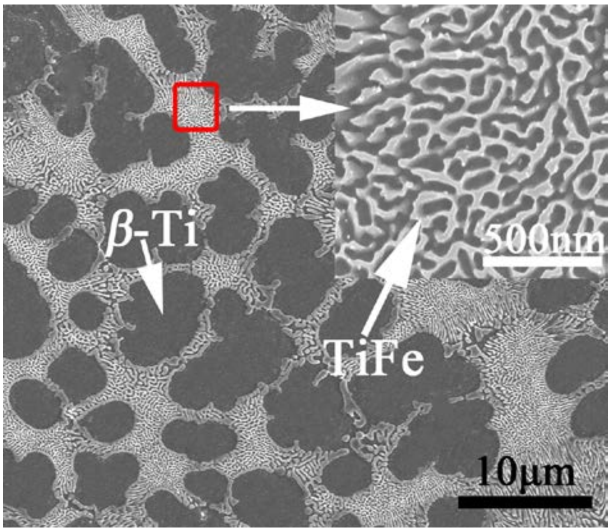

Fig. S2 DSC curves of the 70h-milled $Ti_{62}Nb_{12.2}Fe_{13.6}Co_{6.4}Al_{5.8}$ and $Ti_{66}Nb_{13}Fe_8Co_{6.8}Al_{6.2}$ nanocomposite powder. Compared with single endothermic peak at 1185 °C for the 70h-milled $Ti_{66}Nb_{13}Fe_8Co_{6.8}Al_{6.2}$ nanocomposite powder, two evident endothermic peaks were observed at temperatures of 1125 °C and 1180 °C for the 70h-milled $Ti_{62}Nb_{12.2}Fe_{13.6}Co_{6.4}Al_{5.8}$ nanocomposite powder, respectively, confirming that there exists a semi-solid interval between 1080-1200 °C in this alloy.

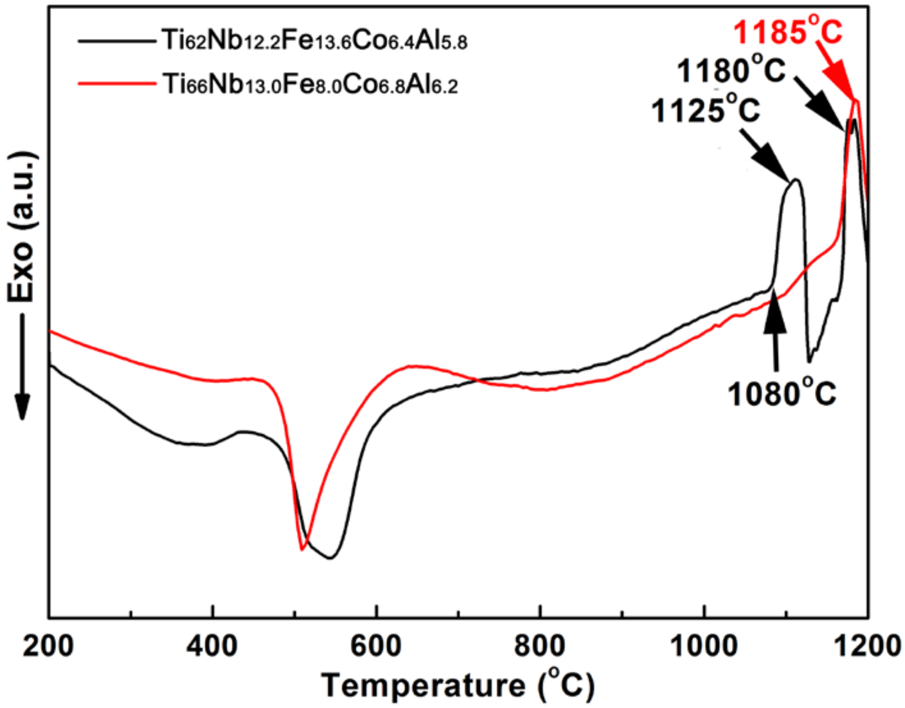

Fig. S3 XRD patterns of the as-milled alloy powder and the as-sintered bulk alloys at different temperatures. "M" denotes $Ti_{62}Nb_{12.2}Fe_{13.6}Co_{6.4}Al_{5.8}$ alloy, and "N" denotes additional $Ti_{66}Nb_{13}Fe_8Co_{6.8}Al_{6.2}$ alloy. The number "X" in "S-X" denotes sintering temperature.

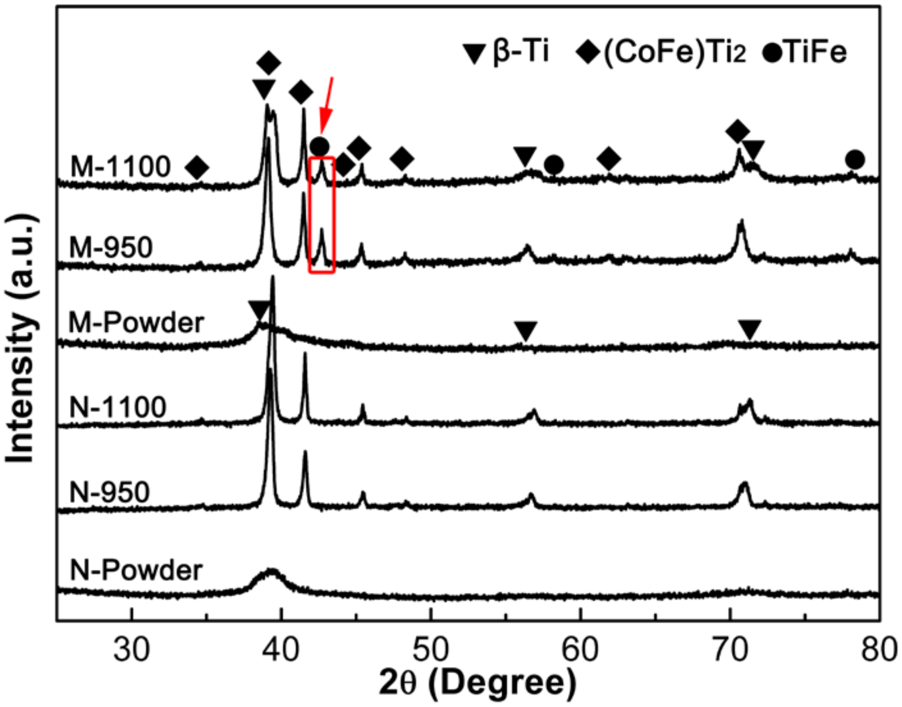

Fig. S3 displays XRD patterns of the $Ti_{62}Nb_{12.2}Fe_{13.6}Co_{6.4}Al_{5.8}$ and additional $Ti_{66}Nb_{13}Fe_{8}Co_{6.8}Al_{6.2}$ alloys sintered at different temperatures. Obviously, the 70h-milled alloy powder M and N are composed mainly of glassy phase and nanocrystalline β-Ti phase, in agreement with the TEM observations (Fig. 1 in the main manuscript) and the DSC analysis (Fig. S2). Comparing to that the sintered N alloys having a two-phase structure with β-Ti and $(CoFe)Ti_2$ phases, all M alloys sintered at different temperatures consist mainly of three-phase structure of TiFe, β-Ti and $(CoFe)Ti_2$ phases. The SEM and TEM observations (Fig. 1 in the main manuscript) further prove this result. Comparing with the sintered M alloys, TiFe phase is not observed in the sintered N alloys at all sintering temperatures, especially at 1100 ^{o}C.

Fig. S4 SEM images of the M and additional N alloys sintered at different temperatures: (a) M-950 ^{o}C, (b) M-1100 ^{o}C, (c) N-950 ^{o}C, and (d) N-1100 ^{o}C, respectively. "M" denotes $Ti_{62}Nb_{12.2}Fe_{13.6}Co_{6.4}Al_{5.8}$ alloy, and "N" denotes additional $Ti_{66}Nb_{13}Fe_{8}Co_{6.8}Al_{6.2}$ alloy. The number "X" in "S-X" denotes sintering temperature.

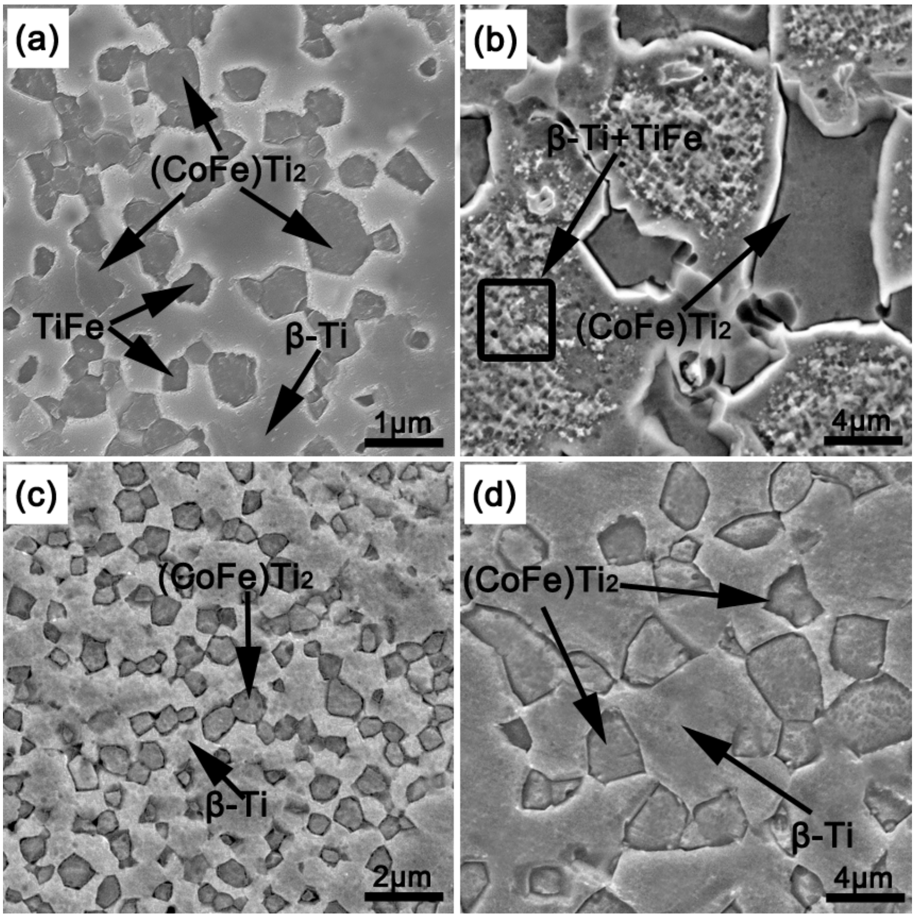

Fig. S4 presents SEM images of the M and N alloys sintered at 950 ^{o}C and 1100 ^{o}C. Evidently, the specimen M-950 displays a microstructure of $(CoFe)Ti_2$ phase and TiFe phase surrounded by β-Ti matrix (Fig. S3(a)), which is in agreement with the TEM observation (Fig. 1d in the main manuscript). With further increasing the sintering temperature, liquid phase begins to form due to the eutectic transformation between the β-Ti and TiFe phases, and then the formed liquid phase with highly dense random-packed structure transforms into nanostructured TiFe and β-Ti matrix in the process of normal solidification, while the remained $(CoFe)Ti_2$ solid phase further grows to micrometer size (Fig. S4(b)). It is confirmed that the formed liquid phase results from the eutectic reaction between the β-Ti and TiFe phases, and the $(CoFe)Ti_2$ phase remains solid state at the sintering temperature of 1100 ^{o}C. This can be proved based on the comparison with the result of the N alloy with lower Fe content. As shown in Fig. S4(c), the sample N-950 exhibits a composite microstructure of $(CoFe)Ti_2$ phase embedded into β-Ti matrix, and EDX analysis shows that the chemical compositions of the $(CoFe)Ti_2$ phase and β-Ti matrix in the sample N-950

are $Ti_{61.9}Nb_{2.7}Fe_{19.8}Co_{13.3}Al_{2.3}$ and $Ti_{64.9}Nb_{22.7}Fe_{5.5}Co_{0.2}Al_{6.7}$, respectively, which are close to those of the $(CoFe)Ti_2$ and β-Ti phases in the alloy M. According to the Co-Ti binary phase diagram, the fcc $CoTi_2$ and bcc β-Ti take an eutectic reaction to transform into liquid phase at 1020 ^oC. However, in the present case, the SEM image of the alloy N-1100 displays a similar microstructure to that of the alloy N-950, where eutectic microstructure was not found in the sample N-1100 (Fig. S3(d)). Besides, melting phenomenon was also not observed in the punch displacement curve of the additional experiment for the alloy N at the sintering temperature of 1100 ^oC, implying that the eutectic reaction between β-Ti and $(FeCo)Ti_2$ phases is more difficult than that in the binary Ti-Co alloy due to the substitution of Fe atoms to Co positions in the fcc $CoTi_2$ compound. It was reported that $(CoFe)Ti_2$ phase can be the primarily precipitated phase from the melt in Ti-Fe-Co alloy during solidification, implying Fe atoms substitute Co positions in fcc $CoTi_2$ compound may result in a higher melting temperature in $(CoFe)Ti_2$ than in TiFe phase and β-Ti phase in the alloy with a given composition [1]. From another point of view, if forming liquid phase originates from the ternary eutectic transformation of the bcc β-Ti, bcc TiFe and fcc $(CoFe)Ti_2$, it is difficult to obtain a bimodal microstructure in the present case according to the formation mechanism of bimodal microstructure in rapid solidification. Therefore, the formed liquid phase can be explained based on the eutectic transformation between the β-Ti and TiFe phases.

Fig. S5 Mould outer photos of the specimen M (a) and additional N (b) sintered at 1100 ^oC. "M" denotes $Ti_{62}Nb_{12.2}Fe_{13.6}Co_{6.4}Al_{5.8}$ alloy, and "N" denotes additional $Ti_{66}Nb_{13}Fe_8Co_{6.8}Al_{6.2}$ alloy. The color of the M alloy is golden yellow as shown in Fig. S5a. This indicates that the alloy surface has been oxidized when the sample was removed from the vacuum sintering furnace of SPS system. But EDX analysis indicates that the sample did not contain oxygen element except surface of sample.

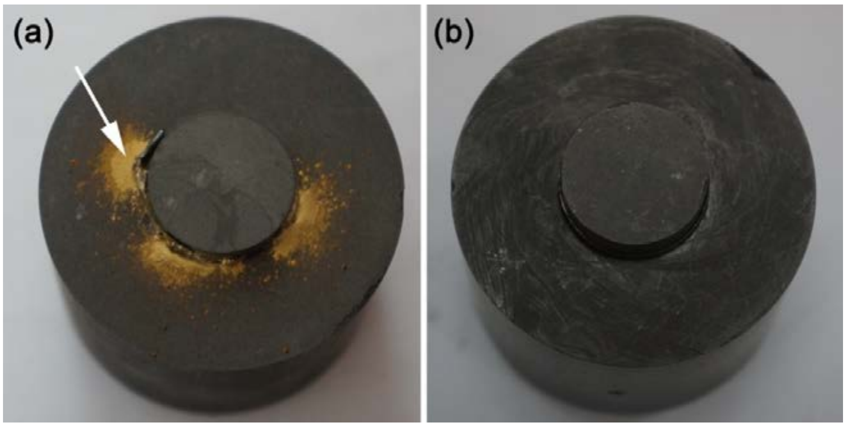

Fig. S5 displays the mould outer photos of the specimen M and N sintered at 1100 °C. Apparently, a small amount of liquid alloy melt was squeezed out from the gap of graphite die in the alloy M-1100 (Fig. S5a) and the ticktack of the squeezed liquid drops with the back plate can be heard during sintering process, indicating local melting has occurred in the alloy M. For the alloy N (Fig. S5b), it is consistent with the SEM observations. No obvious metallic liquid were found on the surface of graphite die. This further confirms that the alloy M possess a lower melting temperature than the alloy N which is composed of only $(CoFe)Ti_2$ and β-Ti phases. The squeezed liquid alloy melt further confirms the presence of a semi-solid interval in the alloy M.

Fig. S6 Shrinkage displacement of the punch versus the sintering temperature for additional N alloy ($Ti_{66}Nb_{13}Fe_8Co_{6.8}Al_{6.2}$) with a lower Fe content. The punch displacement does not display an instantaneous increase around 1080 °C, indicating that the melting phenomenon did not occur in the sintered alloy.

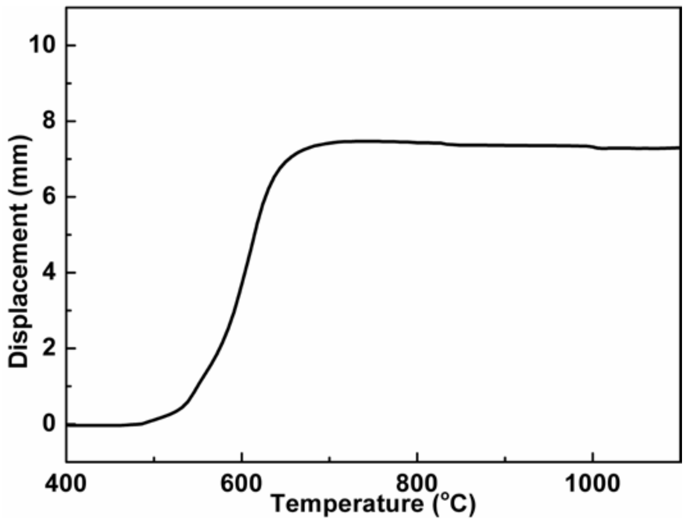

Fig. S7 Cooling rate as a function of the temperature for the alloy M-1100 ("M" denotes $Ti_{62}Nb_{12.2}Fe_{13.6}Co_{6.4}Al_{5.8}$ alloy and 1100 denotes the sintering temperature), noting that the cooling rate is about 400 °C/min at the temperature near 1000 °C. It is close to the cooling rate of conventional casting.

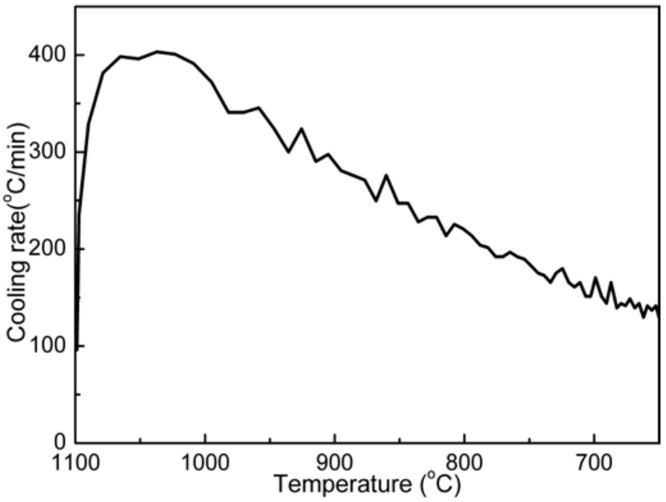

In summary, the semi-solid state induced by the eutectic reaction between the TiFe and β-Ti phases rather than that between the $(CoFe)Ti_2$ and β-Ti phases can also be determined by the additional experiments carried out in $Ti_{66}Nb_{13}Fe_8Co_{6.8}Al_{6.2}$ alloys (N alloy) with lower Fe content compared with present $Ti_{62}Nb_{12.2}Fe_{13.6}Co_{6.4}Al_{5.8}$ alloy (M alloy).

References:

[1] D.V. Louzguine-Luzgin, L.V. Louzguina-Luzgina, H. Kato, A. Inoue, Acta. Mater. 53 (2005) 2009.
